# Enhanced Host-Parasite Resistance Based on Down-Regulation of *Phelipanche aegyptiaca* Target Genes Is Likely by Mobile Small RNA

**DOI:** 10.3389/fpls.2017.01574

**Published:** 2017-09-12

**Authors:** Neeraj K. Dubey, Hanan Eizenberg, Diana Leibman, Dalia Wolf, Menahem Edelstein, Jackline Abu-Nassar, Sally Marzouk, Amit Gal-On, Radi Aly

**Affiliations:** ^1^Department of Plant Pathology and Weed Research, Newe Ya’ar Research Center, Agricultural Research Organization, Volcani Center Ramat Yishay, Israel; ^2^Department of Plant Pathology and Weed Research, Agricultural Research Organization, Volcani Center Rishon LeZion, Israel; ^3^Department of Plant Science, Agricultural Research Organization, Volcani Center Rishon LeZion, Israel

**Keywords:** *Phelipanche*, root parasite, siRNA, VIGS, expression, trans-silencing

## Abstract

RNA silencing refers to diverse mechanisms that control gene expression at transcriptional and post-transcriptional levels which can also be used in parasitic pathogens of plants that Broomrapes (*Orobanche/Phelipanche* spp.) are holoparasitic plants that subsist on the roots of a variety of agricultural crops and cause severe negative effects on the yield and yield quality of those crops. Effective methods for controlling parasitic weeds are scarce, with only a few known cases of genetic resistance. In the current study, we suggest an improved strategy for the control of parasitic weeds based on trans-specific gene-silencing of three parasite genes at once. We used two strategies to express dsRNA containing selected sequences of three *Phelipanche aegyptiaca* genes *PaACS, PaM6PR*, and *PaPrx1* (pma): transient expression using *Tobacco rattle virus* (TRV:pma) as a virus-induced gene-silencing vector and stable expression in transgenic tomato *Solanum lycopersicum* (Mill.) plants harboring a hairpin construct (pBINPLUS35:pma). siRNA-mediated transgene-silencing (20–24 nt) was detected in the host plants. Our results demonstrate that the quantities of *PaACS* and *PaM6PR* transcripts from *P. aegyptiaca* tubercles grown on transgenic tomato or on *TRV*-infected *Nicotiana benthamiana* plants were significantly reduced. However, only partial reductions in the quantity of *PaPrx1* transcripts were observed in the parasite tubercles grown on tomato and on *N. benthamiana* plants. Concomitant with the suppression of the target genes, there were significant decreases in the number and weight of the parasite tubercles that grew on the host plants, in both the transient and the stable experimental systems. The results of the work carried out using both strategies point to the movement of mobile exogenous siRNA from the host to the parasite, leading to the impaired expression of essential parasite target genes.

## Introduction

Parasitic weeds such as broomrapes do not possess functional roots and do not have effective photosynthesis (Parker and Riches, 1993). Instead, they develop special intrusive organs (haustoria) that penetrate crop roots, directly connecting them to the vascular system of the crop plants that serve as their hosts (Joel and Losner-Goshen, 1994; Yoder, 1999; Westwood, 2000). The haustorium is the organ that distinguishes parasitic from non-parasitic plants (Kuijt, 1969). This organ forms the physical and physiological connection between parasite and host, and its interaction with tissues is important for the translocation of molecules and macromolecules (Aly, 2013). By acting as a strong sink relative to the host, broomrapes channel the flow of water, nutrients, and other molecules from the host to themselves, thereby damaging crop development and greatly reducing yields (Joel, 2000). Broomrapes have evolved sophisticated systems for detecting the presence of host plants and coordinating their development with it (Joel and Losner-Goshen, 1994; Yoder, 1999; Bouwmeester et al., 2003). Following successful attachment, broomrape tissues adjacent to the host root grow into a bulbous structure called a tubercle (Kuijt, 1977). After approximately 4 weeks of growth, a floral meristem is produced, which emerges above ground to flower and disseminate seeds. Effective means for the control of broomrapes are few (Aly, 2007).

The best long-term strategy for controlling parasitic weeds may be through the identification and breeding of resistant crop genotypes (Cubero and Hernández, 1991; Ejeta et al., 1991). However, despite many years of work by plant breeders, only a handful of resistant crop cultivars are currently available.

Plants have evolved a variety of gene-silencing pathways mediated by small RNA sequences (siRNA), which are 21 or 24 nt in size. siRNA suppresses the expression of sequence-homologous genes at the transcriptional and post-transcriptional levels (Baulcombe, 2004; Ding, 2010). The production of hairpin RNA (hpRNA) in transgenic plants is a powerful tool for suppressing gene expression in plants (Wesley et al., 2001; Mansoor et al., 2006; Bandaranayake and Yoder, 2013) through a process known as post-transcriptional gene-silencing (PTGS).

Gene-silencing in plants has been shown to effectively control nematodes (Atkinson et al., 2003; Huang et al., 2006) and viruses (Prins et al., 2008; Leibman et al., 2015), and evidence is available to suggest a natural antiviral role for RNA silencing in vertebrates, fungi, worms, and flies (Fire et al., 1998; Denli and Hannon, 2003). RNAi strategies have also been tried for the control of parasitic plants such as *Triphysaria pusilla* (Benth.) T.I. Chuang and Heckard (Tomilov et al., 2008), *Striga hermonthica* (Delile) Benth. (de Framond et al., 2007; Ejeta and Gressel, 2007), *Phelipanche aegyptiaca* (Pers.) Pomel (Aly et al., 2009), and *Cuscuta pentagona* Engelm. (Alakonya et al., 2012).

Virus-induced gene-silencing (VIGS) is an RNA silencing-based technique used for the targeted down-regulation of a host gene, to allow the analysis of the function of that gene (Lu et al., 2003). It has also been used to silence a wide variety of genes in plants (Robertson, 2004). VIGS-derived dsRNA can be transferred from a host plant to herbivores (Kumar et al., 2012) and parasitic plants and suppress the expression of target genes (Aly et al., 2014). Using the VIGS technique, we have shown that transient knock-down of *Phelipanche aegyptiaca CCD7* and *CCD8* inhibits the development of parasite tubercles and the infestation process in tomato host plants (Aly et al., 2014).

The specific genes selected for silencing are genes that play critical roles in the life cycle of the parasite: 1-amino-cyclopropane-1-carboxylate synthase (*PaACS* synthase; accession no. AB219097) is a key regulatory enzyme in the ethylene biosynthetic pathway, which delays the flowering of the parasite growing on transgenic plants (Trusov and Botella, 2006). Mannose 6-phosphate reductase (*PaM6PR*; Aly et al., 2009) regulates mannitol content in *P. aegyptiaca*; mannitol is essential for the movement of water and nutrients from the host to the parasite (Everard et al., 1997; Delavault et al., 2002). Peroxidase (*PaPrx1*; accession no. AY692263) plays an important role in mediating the parasite’s responses and signals during the early stages of infection (Keyes et al., 2001). It may also play a role in the infection process or in the development of the parasite, or even loosen the host cell wall (Foreman et al., 2003; Liszkay et al., 2004), thereby facilitating penetration.

We used two silencing strategies to degrade the RNA of three important *P. aegyptiaca* genes and demonstrated that levels of endogenous *PaACS* synthase and *PaM6PR* transcripts from *P. aegyptiaca* tubercles grown on transgenic or tobacco rattle virus (TRV)-mediated *Nicotiana benthamiana* plants were significantly reduced and significantly inhibited the development of the parasite.

## Materials and Methods

### Selection of the Candidate Genes, Vector Construction and Generation of Transgenic Plants

Three *P. aegyptiaca* genes, *PaPrx1*, *PaM6PR*, and *PaACS*, were selected to be knocked down. The sequences of *PaPrx1* (AY692263), *PaM6PR* (Aly et al., 2009), and *PaACS* (AB219097) of *P. aegyptiaca* were fished out from NCBI and confirmed with the PPGP website.^1^ The unique and non-homologous sequences of respective candidate genes were selected as described by Aly et al. (2009). RNA was isolated from *P. aegyptiaca* tubercles (Spectrum^TM^ Plant Total RNA Kit, STRN50, Sigma) and then used to prepare cDNA (Verso cDNA kit, AB-1453/A, Thermo) for the further amplification of selected sequences. The selected region of *PaPrx1* cDNA was amplified using the forward primer 5′-CGAGCTCCCAAGCAATTAAGTTTAGTG-3′ and the reverse primer 5′-GGGGTACCCCTCTCACGTGATATTGC-3′, flanking the *SacI* and *KpnI* sites, respectively, and cloned into pUC19. The selected region of the *PaM6PR* gene was amplified using the forward primer 5′-GGGGTACCTCCAATGAGGATATGGAACTG-3′ and the reverse primer 5′-GCGTCGACGAGGTTGGAAGAGAACAATAC-3′, flanking the *KpnI* and *SalI* sites, respectively, and fused to the *PaPrx1* gene in the recombinant pUC19. The selected fragment of *PaACS* was amplified using the forward primer 5′-GCGTCGACTTGATGACGATCGAGTGGCG-3′ and the reverse primer 5′-CCCAAGCTTATTTGCGGGCCAGCTGGAG-3′, flanking *SalI* and *HindIII*, respectively, and cloned in recombinant pUC19 containing both parts of the *PaPrx1* and *PaM6PR* genes. The recombinant clones containing the three fused genes were confirmed with restriction analysis and nucleotide sequencing. For the VIGS assay, the pma sequences (including the three segments of the parasite genes) were amplified using the forward primer 5′-GCGGCCGCTCTAGACCAAGCAATTAAGTTTAGTG-3′ and the reverse primer 5′-CCGCTCGAGGGATCCATTTGCGGGCCAGCTGG-3′, and then cloned into the pTRV2 vector at the *XbaI* and *BamHI* sites.

The above three fragment genes (799 nt) were also cloned in a hairpin configuration in a binary vector pBINPLUS under the control of the CaMV35S promoter, as described by Leibman et al. (2015). The transgenic tomato plants were generated using kanamycin as a selection marker (Barg et al., 1997; Leibman et al., 2015). T1 seeds were collected and sterilized with 70% ethanol for 1 min and 1% sodium hypochlorite and 0.01% Tween for 15–20 min, and then rinsed three times with sterilized distilled water. Ten seeds from each of the tomato T1 lines were grown on media (20 g sucrose, 4.4 g MS, pH 5.8, with 6 g phytagel) containing 100 μg/ml kanamycin. The petridishes containing the seeds were kept in a dark growth chamber for 3 days. After that period, the transgenic seedlings were transplanted into pots in a greenhouse.

### RNA Isolation, RT-PCR, and qRT-PCR Analysis

Total RNA was extracted from 5 to 10 mm *P. aegyptiaca* tubercles grown on silenced and non-silenced *N. benthamiana* plants, and tubercles attached to transgenic tomato and control tomato plants. First-strand cDNA was synthesized using 1 μg of total RNA extracted from *P. aegyptiaca* tubercles. The quantitative reaction was performed using an ABI-Prism 7000 Real-Time PCR Detection System (Applied Biosystems) and SYBR Green Master Mix (Thermo-AB4162) according to the manufacturer’s protocol. For real-time experiments, we used the following primers: for the *PaACS* gene, forward 5′-GGGCATGGTGGGTATTTGC-3′ and reverse 5′-TACTATGTGAGAATCTTGGGCTTGA 3′; for *PaM6PR*: forward 5′-CCAATGAGGATATGGAACTGTTGA-3′ and reverse 5′-CATGGGAGAGAAACTTATGCGAAAA-3′; and for *PaPrx1*: forward 5′ ATCCATCAACTTTGTTGCTGTGA-3′ and reverse 5′-ACGACATGTGCGAGAGTAGAATG-3′. Expression of the candidate target genes was normalized to the expression of the *Actin* gene using the forward primer 5′-ATGGGCCAGAAAGATGCATATGTT-3′ and the reverse primer 5′-GTGTGATGCCAAATTTTCTCCATGT-3′. Relative gene expression was calculated using the 2^-ΔΔCt^ method (Livak and Schmittgen, 2001). The qRT-PCR reaction conditions were as follows: 15 min activation at 95°C, followed by 40 cycles of 95°C for 10 s, 60°C for 15 s, and 70°C for 20 s. The qRT-PCR experiment was performed in biological and experimental duplicates using PCR Master Mix (cat.42-138; Apex^TM^).

### siRNA Analysis in Transgenic Roots and in VIGS-Assayed Plants

Total RNA was isolated from roots of transgenic tomato plants using an EZ-RNA II kit (Total RNA Isolation Kit, 20-410-100, Biological Industries). Expression of transgene siRNA were detected by northern blot analysis using a cDNA-transcript probe harboring the three target genes (^32^P-labeled cDNA probe) according to a standard protocol (Aly et al., 2009).

### VIGS Assay

Tobacco rattle virus-virus-induced gene-silencing experiments were performed with *N. benthamiana* seedlings that had 3–4 leaves. Agro-infiltration of tobacco leaves were performed as described by Bachan and Dinesh-Kumar (2012). In brief, the following plasmids: TRV-RNA1 (TRV), TRV-RNA2 (pTRV2:*PaPrx1-PaM6PR-PaACS*, abbreviated TRV:pma), and empty TRV were transformed with *Agrobacterium* strain EHA105 using standard protocols. Single colonies were inoculated for primary broth culture (5 ml), followed by secondary broth culture (50 ml) in the presence of suitable antibiotics. The colonies were then grown overnight at 28°C. The next day, 50 ml of cell culture was pelleted by centrifugation at 3000 rpm for 15 min. The recovered pellet was dissolved in infiltration medium (10 mM MES; 10 mM MgCl_2_; 250 μ M acetosyringone in double-deionized water) adjusted to an OD of 1.0 (600 nm), and then incubated at room temperature for 3 h. *Agrobacterium* was introduced into the lower surface of the tobacco leaf with a 2.0 ml syringe. Just before infiltration, a culture of TRV and TRV:pma in a 1:1 ratio was prepared. RNA was isolated from TRV- and TRV:pma-infected leaves and roots 10 days after infiltration and then subjected to RT-PCR analysis. The expression analyses of TRV and TRV:pma were performed using the following primers: pTRV1 forward: 5′-CCTTTGAACGCGGTAGAACG-3′, pTRV1 reverse: 5′-TGCAGAGCAGGAACTCTATC-3′ and pTRV2 forward: 5′-TTACGGACGAGTGGACTTAG-3′ and pTRV2 reverse: 5′-CTATGGTAAGACAATGAGTCG-3′.

### Evaluation of Plant Resistance to the Parasite

*Phelipanche aegyptiaca* seeds were collected from an infested tomato field in the Bet She’an Valley in eastern Israel. *N. benthamiana* plants were used as hosts for TRV infection. Host plants were transplanted into 2.0-L pots filled with soil (light-medium clay with 63% sand, 12% silt, and 22% clay) and grown in a greenhouse under natural light with an average 14 h of daylight and a temperature of 20 ± 6°C. The plants were watered and fertilized as needed. *N*. *benthamiana* seedlings were transplanted into pots containing soil infested with *P. aegyptiaca* seeds (20 mg), 7–10 days before agro-infiltration. Host roots from TRV-VIGS and control plants were collected 25–30 days after they were first exposed to the *P. aegyptiaca* seeds. *P. aegyptiaca* tubercles larger than 2 mm were counted and weighed and their RNA was then isolated.

The host resistance of transgenic tomato lines (T_1_) was evaluated by challenging the host plants with the parasite seeds in 2.0-L pots. The roots were washed after the parasite inflorescences emerged above the ground. *P. aegyptiaca* tubercles larger than 2 mm were counted and weighed, and RNA was then isolated from those tubercles for the analysis of target gene expression. Host and parasite morphology and biomass were measured as described by Aly et al. (2009).

### Peroxidase Assay

Peroxidase activity was evaluated using the Amplex^®^ Red Hydrogen Peroxidase/Peroxidase Assay Kit (A22188, Molecular Probes). Briefly, 200 mg of the parasite tubercles attached to transgenic and non-transgenic host roots were collected and frozen in liquid nitrogen, and then homogenized in 1.2 ml of 0.2 M potassium phosphate buffer (pH 7.8). Samples were centrifuged at 15000 rpm for 20 min at 4°C. The supernatants were stored and the pellet was re-suspended again in 0.8 ml of the same buffer followed by centrifugation. Both supernatants were combined and stored on ice, and used to determine peroxidase activity.

## Results

### *P. aegyptiaca* Target Gene Sequences

Based on the database of *P. aegyptiaca* ESTs from the Parasitic Plant Genome Project^2^ (Westwood et al., 2011), PubMed-NCBI data sequences and an older database of *P. aegyptiaca* sequences (Aly et al., 2009), we identified and confirmed suitable DNA sequences (Supplementary Figure S1) from non-homologous regions of the three target genes that differ between *P. aegyptiaca*, tomato and *N. benthamiana*, to avoid silencing any host genes.

### Silencing of *P. aegyptiaca* Target Genes via TRV Vectors

**FIGURE 1:**
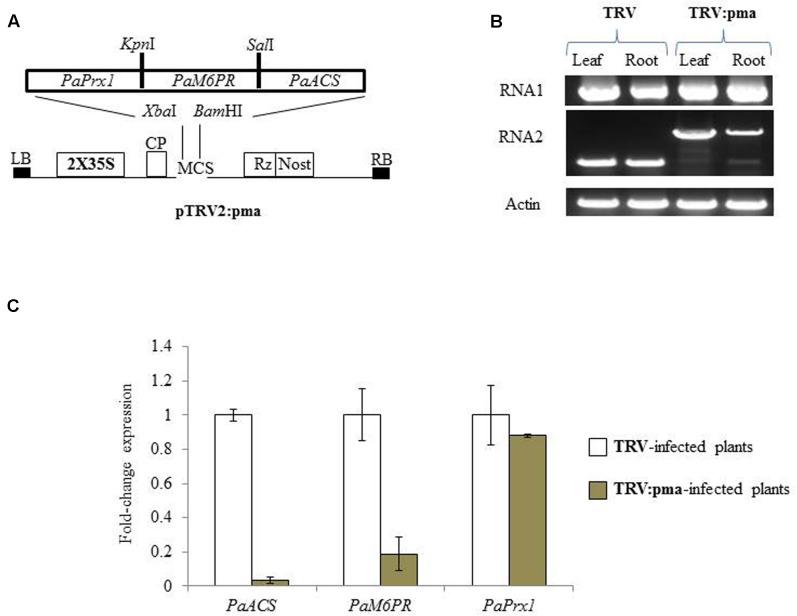
Suppression of *PaACS*, *PaM6PR*, and *PaPrx1* mRNA in *Phelipanche aegyptiaca* via Tobacco rattle virus-virus-induced gene-silencing (TRV-VIGS) assayed in *Nicotiana benthamiana* plants. **(A)** Schematic representation of the construct cloned in the pTRV2 vector according to Liu et al. (2002). **(B)** Systemic infection of recombinant TRV:pma and the TRV control in leaves and roots of *N. benthamiana* plants. RT-PCR was used to assess the accumulation of TRV RNA1 and 2. The actin gene served as a control. **(C)** Quantification of *PaACS*, *PaM6PR*, and *PaPrx1* mRNA by qRT-PCR analysis was normalized to actin transcript levels in *P. aegyptiaca* tubercles attached to *N. benthamiana* plants that were infected with TRV and TRV:pma. All analyses were performed using three biological replicates. TRV-infected plants were calibrated to the value 1.

The selected target regions of *PaM6PR* (268bp), *PaACS* (299bp), and *PaPrx1* (232bp) from *P. aegyptiaca* (Supplementary Figure S1) were cloned in a transient expression system (TRV) vector (**Figure 1A**), as described by Liu et al. (2002). *N. benthamiana* plants were agro-infiltrated with the recombinant TRV2:pma and TRV (**Figure 1A**) according to the method described by Bachan and Dinesh-Kumar (2012). Accumulation of TRV and TRV:pma in roots and leaves of *N. benthamiana* plants was confirmed by RT-PCR (**Figure 1B**). The expression levels of the target gene mRNA in *P. aegyptiaca* grown on assayed *N. benthamiana* plants were evaluated using quantitative RT-PCR. This analysis showed that the transcript amounts of *PaACS* and *PaM6PR* were significantly reduced in the parasite tubercles growing on *N. benthamiana* plants infected with recombinant TRV as compared to *N. benthamiana* plants infected with TRV (**Figure 1C**). No significant suppression of the production of *PaPrx1* transcripts was observed in the parasite tubercles grown on *N. benthamiana* plants infected with recombinant TRV (**Figure 1C**).

### Retardation of *P. aegyptiaca* Development on *N. benthamiana* Plants Infected with TRV:pma

**FIGURE 2:**
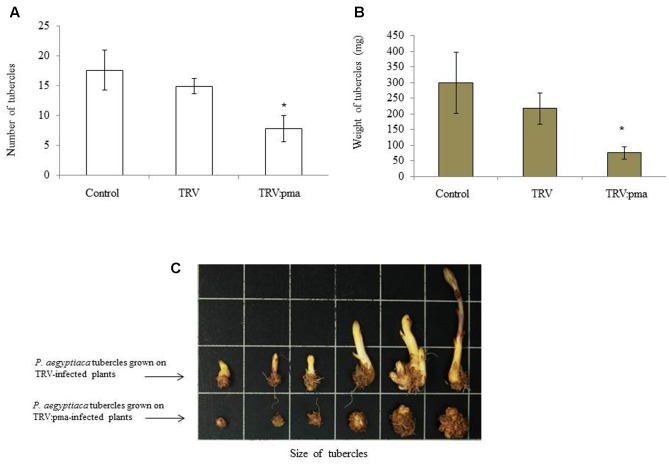
Retardation of *P. aegyptiaca* development on VIGS-assayed *N. benthamiana* plants. The resistance of *N. benthamiana* plants to *P. aegyptiaca* was assayed by transplanting *N. benthamiana* seedlings into pots containing soil infested with *P. aegyptiaca* seeds (20 mg) 7–10 days before agro-infiltration. To evaluate host resistance to the parasite, host roots of rec-TRV- and TRV-treated plants were rinsed 25–30 days after they were challenged with *P. aegyptiaca* seeds. Tubercles larger than 2 mm (Diameter) were considered for analysis. The number of parasitic tubercles **(A)**, average weight of tubercles **(B)**, and representative tubercle growth of the parasitic plants attached to rec-TRV- and TRV-treated plants **(C)** were analyzed. Bars represent means of 10 replicates and vertical lines indicate SE values. Asterisks (^∗^) indicate means different from that of the control and significant differences between empty vector (EV)-infiltrated plants and vector-containing target genes in the VIGS trials, as determined by Student’s *t*-test, α = 0.05.

*Nicotiana benthamiana* plants were assayed for resistance to *P. aegyptiaca* in pots in which infected plants were pre-challenged with the parasite seeds 10 days before agro-infiltration in the greenhouse (Aly et al., 2014). Parasite infection rates and the number and total weights of *P. aegyptiaca* tubercles larger than 2 mm were determined on TRV:pma and TRV control plants 2 weeks after agro-infiltration. TRV:pma-treated plants expressing the target sequences of *PaACS*, *PaM6PR*, and *PaPrx1*had significantly fewer parasite tubercles and the weight of those tubercles was also more than 50% lower among these plants, as compared to the control plants (**Figures 2A,B**). Growth of the parasite shoots also ceased (**Figure 2C**). Our data suggest that mobile siRNA might move from the host plant to the parasite tubercles and differentially affected the silencing of the target genes.

Since the efficacy of the trans-silencing of the target sequences in *P. aegyptiaca* was confirmed for at least two genes (*ACS* and *M6PR*) through the use of the transient VIGS strategy, we conducted experiments for stable transformation into tomato *Solanum lycopersicum* L.‘MP-1’plants, to determine efficacy of this trans-silencing strategy in stable transgenic lines.

### Characterization of Stable Transgenic Tomato Lines and Their Resistance to the Parasite

**FIGURE 3:**
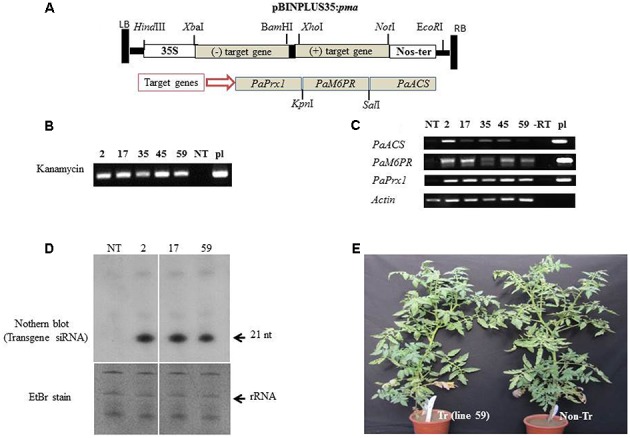
Integration and expression of the *PaACS*, *PaM6PR*, and *PaPrx1* fragments in T1 transgenic tomato lines. **(A)** Schematic representation of the silencing construct pBINPLUS35S*:pma* binary vector harboring the target genes *PaACS*, *PaM6PR*, and *PaPrx1* in hairpin configuration. **(B)** The presence of the transgene in selected T1 lines (2, 17, 35, 45, and 59) was confirmed by PCR analysis of extracted DNA. Lanes NT and pl show the PCR products from the non-transgenic control plants and the pBINPLUS35S:*pma* binary vector, which served as a positive control. For RT-PCR analysis, total RNA was extracted from tomato roots and cDNA was then prepared using random hexamer primers. **(C)** Levels of the transgene transcripts were analyzed by RT-PCR of the self-pollinated progenies (T1) of the transgenic lines 2, 17, 35, 45, and 59. Expression of the actin gene was used as a control for the RT-PCR procedure, the construct pBINPLUS35S*:pma* (pl) served as a positive control and (–RT) served as a negative control. **(D)** Northern blot analysis of transgene-siRNA (t-siRNAs) accumulated in transgenic lines 2, 17, and 59. Non-transformed tomato (NT) served as a negative control. Approximately 30 μg of total RNA from each sample were separated on a 15% urea-PAGE gel and then transferred to a nylon NX membrane. Hybridization was performed with ^32^P-labeled transcripts of the transgene clone. The gel was stained with ethidium bromide for RNA evaluation prior to transfer to nylon (EtBr stain). **(E)** Growth and appearance of the transgenic tomato plants (Tr) and non-transgenic (non-Tr) tomato plants in a greenhouse.

The binary pBINPLUS35:*pma* construct (**Figure 3A**) harboring fragments of *PaPrx1*, *PaM6PR*, and *PaACS* in a hairpin configuration was transformed into tomato *Solanum lycopersicum* ‘MP-1’as described by Leibman et al. (2015). Several independent lines of transgenic tomato containing *pBINPLUS:pma* were developed through *Agrobacterium*-mediated transformation. Twenty-six independent transgenic tomato lines were generated and five lines (2, 17, 35, 45, and 59) were selected for use in further experiments based on transgene expression as determined by RT-PCR. PCR was used to confirm the presence of the transgene in the selected T1 transgenic lines (**Figure 3B**). A segregation ratio of close to 3:1 was noted for kanamycin resistance (data not shown), which may indicate the presence of a single locus in those transgenic lines. Expression levels of the transgene transcripts of lines 2, 17, 35, 45, and 59 (T1 progeny) were analyzed by RT-PCR, using target gene-specific primers (**Figure 3C**).

The transgene transcripts were detected only in the transgenic lines using specific primers (**Figure 3C**). Interestingly, the level of transgene transcript varied between the different target genes: *Prx1* ≥*M6PR* ≥*ACS*. This could be due to the orientation and position of the fragment in the inverted repeat construct, as was demonstrated by Wroblewski et al. (2014). In addition, the differences in transcript levels (**Figure 3C**) may reflect rapid processing of transgene dsRNA by DCLs to siRNA. So the low level of transcripts in line 59 may indicate a rapid processing to siRNA. This could explain why the low level of transcript in line 59 is associated with more efficient silencing.

To verify the transgene dsRNA processing by DICERs, we used northern blotting to analyze the accumulation of transgene siRNA in the roots of transgenic and non-transgenic lines. The accumulation of transgene siRNA was detected and confirmed in several lines, including lines 2, 17, and 59 (**Figure 3D**). The horticultural traits of the transgenic T1 tomato lines appeared normal and the plants were fertile under greenhouse conditions. No phenotypic differences were observed between these plants and the corresponding non-transformed MP-1 plants during the vegetative (**Figure 3E**) or reproductive growth stages (data not shown).

**FIGURE 4:**
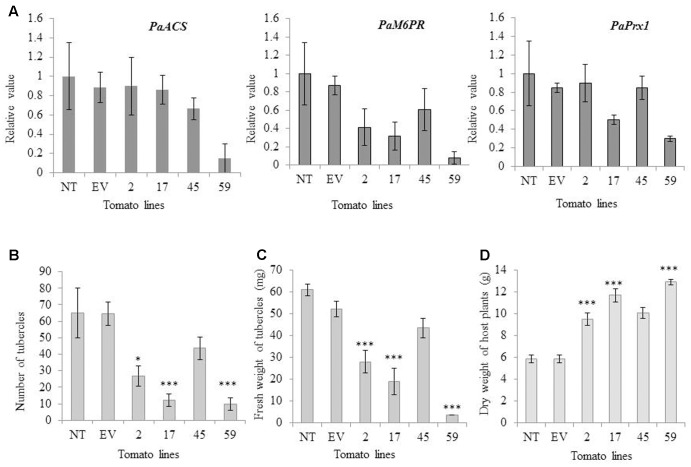
mRNA levels of *PaACS*, *PaM6PR*, and *PaPrx1* in *P. aegyptiaca* tubercles and resistance of transgenic and non-transgenic lines to the parasite. **(A)** Quantification of *PaACS*, *PaM6PR*, and *PaPrx1* mRNA levels by qRT-PCR normalized to equal levels of actin transcripts in the underground tubercles of *P. aegyptiaca* including controls and transgenic tomato plants. Total RNA was extracted from 0.5 g of 3 to 5 pooled *P. aegyptiaca* tubercles grown on five transgenic T1 tomato plants (lines 2, 17, 45, and 59), a non-transgenic control plant (NT) and transgenic plants carrying an EV. Quantitative RT-PCR analysis was performed using primers specific for *PaACS*, *PaM6PR*, and *PaPrx1*. The data presented are relative values calculated following normalization to *P. aegyptiaca* actin with the 2^-ΔΔct^ program. The data are the means of three biological replicates. Bars represent the standard errors of three independent measurements. The graphs in panels **(B,C)** show the number and fresh weights of *P. aegyptiaca* tubercles attached to the transgenic and non-transgenic tomato plants in the greenhouse pot assay. *P. aegyptiaca* tubercles were collected from five transgenic T1 tomato plants (lines 2, 17, 45, and 59), a non-transgenic control plant (NT) and transgenic plants carrying an EV. Means ± SE were calculated based on 10 independent plants. For both experiments, ^∗^ and ^∗∗∗^ indicate means different from NT and EV as determined by Student’s *t*-test at α = 0.05 and α = 0.001, respectively. **(D)** Dry weights(g) of host plants were obtained as described by Hamamouch et al. (2005). Means ± SE were calculated based on 10 independent plants. For both experiments, ^∗^ and ^∗∗∗^ indicate means significantly different from NT and EV as determined by Student’s *t*-test at α = 0.05 and α = 0.001, respectively. (The weight in graph C and D represent individual average amount of each line).

To determine whether transgene siRNA produced in the host would move into *P. aegyptiaca* and affect the accumulation of the parasite mRNA targets, we examined the expression levels of the target genes (*PaPrx1*, *PaM6PR*, and *PaACS*) in viable *P. aegyptiaca* tubercles. Our quantitative RT-PCR analysis showed that the level of endogenous target mRNA in the parasite tubercles was reduced relative to the levels in *P. aegyptiaca* tubercles grown on transgenic T1 tomato plants containing an empty vector (EV) or non-transgenic tomato plants (NT; **Figure 4A**).

Levels of *PaACS*, *PaM6PR*, and *PaPrx1* mRNA in *P. aegyptiaca* tubercles attached to plants of line 59 were significantly suppressed (more than 6-, 12- and 3-fold, respectively; **Figure 4A**). Significant mRNA suppression of *PaM6PR* and *PaPrx1* was observed among plants of line 17 and, among the plants of line 2, only *PaM6PR* mRNA was significantly suppressed (**Figure 4A**).

The resistance of the best candidate lines (2, 17, 45, and 59) to parasite development was evaluated in pot experiments. *P. aegyptiaca* infestation was examined in three separate experiments, which each included 10 biological replicates. To evaluate the resistance of the transgenic lines, we considered and counted only fresh and viable parasite tubercles. Our results indicate that the number of attached parasite tubercles was decreased significantly relative to the non-transgenic plants: 7-fold in line 59, 5-fold in line 17, and more than 2-fold in line 2 (**Figure 4B**). The fresh weights of parasite tubercles and shoots attached to lines 2, 17, and 59 were also significantly lower than those of the parasite tubercles and shoots attached to the control plants (**Figure 4C**). Dry weights of transgenic tomato shoots were significantly higher for lines 2, 17, and 59, as compared to the non-transgenic control plants (**Figure 4D**).

**FIGURE 5:**
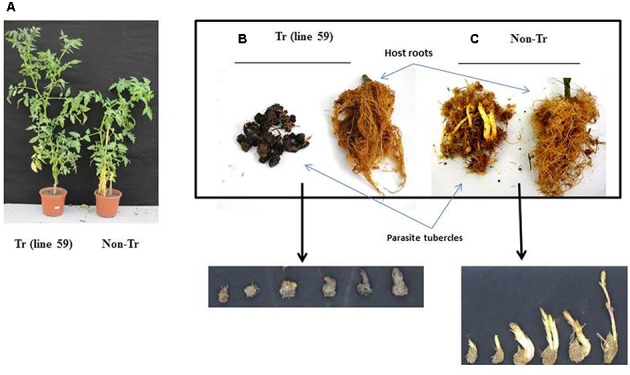
Phenotypes of transgenic tomato plants and *P. aegyptiaca* tubercles grown in pot experiment in the greenhouse. **(A)** Growth and appearance of a representative transgenic tomato plant Tr (line 59) and a representative non-transgenic (non-Tr) plant. **(B)** Roots of transgenic line Tr (line 59) and the parasite tubercles originated from this line. **(C)** Roots of non-transgenic (non-Tr) and the parasite tubercles originated from this line.

The resulting plants appeared normal and were fertile (**Figure 5A**). When grown in soil inoculated with *P. aegyptiaca*, transformed tomato lines 2, 17, 45, and 59 had significantly higher biomass accumulation than non-transgenic tomato lines (**Figure 5A**). Additionally, the transformed plants had higher proportions of necrotic and dead tubercles (**Figure 5B**), as compared to the non-transformed plants (**Figure 5C**). Specifically, the mean proportion of necrotic tubercles on non-transformed plants was 1%; whereas among the transgenic lines 2, 17, 45, and 59, the proportion ranged from 50 to 90%.

## Discussion

As described previously (Aly, 2007; Joel et al., 2007, 2013), parasitic weeds are difficult to control by conventional means due to their life style; they live in close association with the host roots and are concealed underground and out of sight until they have already inflicted irreversible damage. In this study, our hypothesis was based on our previous results showing partial silencing of a single gene (*M6PR*) in *P. aegyptiaca* (Aly et al., 2009). In order to increase the robustness of this resistance, we pyramided multiple hairpin sequences into single vector. We assumed that resistance to *P. aegyptiaca* in tomato would be improved by expressing dsRNA of multiple gene sequences involved with essential regulatory pathways in the parasite. We selected three genes that are important for the parasite’s metabolism (*PaACS*, *PaM6PR*, and *PaPrx1*) for silencing. Suitable DNA fragments (Supplementary Figure S1) from non-homologous regions of the target genes that differ between *P. aegyptiaca* and tomato (to prevent silencing of endogenous genes in the host) were used.

To evaluate host resistance to parasitism by *P. aegyptiaca*, we used two different strategies to knock out three parasite target genes: VIGS and hairpin silencing. Using a VIGS system, we were able to knock-down two candidate genes, *PaACS* and *PaM6PR* (**Figure 1C**), but not *PaPrx1* (**Figure 1C**) in parasite tubercles attached to the host. However, in a stable transgenic system (hairpin-silencing strategy), significant suppression of *PaPrx1* transcripts was observed in the parasite tubercles attached to the roots of transgenic tomato plants of lines 17 and 59 (**Figure 4A**). The lacks of *PaPrx1* transcript suppression in the transient transformation system can be explained by the instability or inefficiencies of *PaPrx1*-siRNAs derived from TRV. Additionally, it might be that the siRNA itself was unable to reach the target gene due to its localization in the tissue.

It is also possible that the targeted *PaPrx1*genes is redundant with other members of their gene family compensating for the silenced genes. This assumption was confirmed, by a nucleotide blast of the selected *PaPrx1* region with the transcriptomic data of *P. aegyptiaca* ESTs from the Parasitic Plant Genome Project^3^ at different developmental stages. Indeed, several members of *PaPrx1* were expressed during the early stages of host infection by the parasite (Supplementary Table S1).

We also measured peroxidase activity in tubercles attached to the roots of selected transgenic plants and found no significant suppression of peroxidase activity in either the VIGS system or the hairpin-silencing system (Supplementary Figure S2), with the exception of line 45, in which peroxidase activity was not correlated with the transcript level (**Figure 4A**). The observed peroxidase activity probably reflects the activity of multiple peroxidases rather than the targeted sequence of *PaPrx1*. Nevertheless, low-level suppression of the *PaPrx1* target gene did not affect the number or weight of the parasite tubercles that developed on the assayed plants treated with the silencing construct (TRV:pma sequences). The number and weight of the tubercles on these host plants were significantly lower than those observed for the control treatment (TRV), as a result of *PaACS* and *PaM6PR* silencing (**Figures 2A,B**). Additionally, the parasite tubercles that developed on the VIGS-assayed plants were small and necrotic and developed abnormally (**Figure 2C**). Accumulation of M6PR siRNA in transgenic tomatoes was shown to correlate with decreased levels of M6PR mRNA (Aly et al., 2009). A similar correlation was previously observed between the accumulation of siRNA and virus resistance (Bucher et al., 2006).

We assume that a silencing signal (i.e., mobile siRNA) travels a long distance (Molnar et al., 2011) and can move from host to parasite through haustoria and targeted the tubercles of the parasite genes. Such long-distance movement of mobile siRNA has also been observed between host plant tissue and the parasites *Triphysaria* (Tomilov et al., 2008) and *Phelipanche* (Aly et al., 2009). Although, TRV-VIGS strategy is based on transient expression and does not rely on transformation, it offers a tremendous advantage to analyze gene functions. Promising target genes identified by TRV-VIGS might be used for stable transformation. Therefore, for stably transformed tomato plants, constructs containing the selected sequences (*PaACS, PaM6PR*, and *PaPrx1*) from *P. aegyptiaca* were cloned into the pBINPLUS plasmid in hairpin configuration, as illustrated in **Figure 3A** and introduced into tomato [*S. lycopersicum* (Mill.)] plants. The presence of the transgenes in transgenic plants was verified by PCR and RT-PCR (**Figures 3B,C**). The accumulation of a large amount of siRNA in the transgenic host plants (2, 17, and 59; **Figure 3D**) could explain the significant reductions in the mRNA levels of the parasite tubercles grown on those transgenic plants. Other transgenic lines (data not shown) had lower amounts of siRNA, reflecting lower levels of mRNA targeting of the parasite.

Silencing efficiency could be related to the amount of siRNA in the plant tissue or to the specific selected sequences of the target genes (Dunoyer et al., 2005). It is also possible that the efficiency of the haustorial connection varied between the different host lines. Silencing variability was shown for GUS activity in GUS-expressing *Triphysaria* that was grown on transgenic lettuce expressing GUS (Tomilov et al., 2008). In our study, the transgenic plants expressing the selected parasite genes were similar in appearance to non-transformed plants (**Figure 3E**), suggesting that the parasite target genes are not detrimental to the host.

Our results demonstrate differential silencing efficacy of the siRNA on target endogenous (*PaACS*, *PaM6PR*, and *PaPrx1*) mRNA from *P. aegyptiaca* tubercles grown on transgenic lines (2, 17, 45, and 59) (**Figure 4A**). For example, the quantity of *PaACS* transcript was significantly reduced in only in one line (line 59), the quantity of *PaM6PR* transcript was significantly reduced in three lines (2, 17, and 59), and the quantity of *PaPrx1* transcript was significantly reduced in two lines (17 and 59; **Figure 4A**).

Differences in the transcript levels or efficiency of mRNA silencing among the different transgenic lines could be related to the amount of siRNA in the host plant tissue or possibly to the efficiency of the haustorial connection to the host lines. In our previous study, accumulation of M6PR siRNA in transgenic tomatoes was shown to correlate with decreased levels of M6PR mRNA (Aly et al., 2009). Recently, the up-regulated expression of *SHOOT MERISTEMLESS-like* (*STM*) home box transcription factors was demonstrated during haustoria formation in *Cuscuta* (Alakonya et al., 2012). That study of transgenic tobacco expressing siRNA of *STM* specific to *Cuscuta* reported the reduced efficacy of dodder infection on transgenic tobacco plants and defects in haustorial connection, development, and establishment on the host (Alakonya et al., 2012).

In the current study, the transgenic lines (2, 17, and 59) had significantly fewer tubercles on their roots and the weight of those tubercles was also significantly reduced, as compared the control plants (**Figures 4B,C**). The transgenic plants (2, 17, 45, and 59) accumulated more biomass than both the non-transformed plants and the plants transformed with an empty vector in the presence of the parasite (**Figure 4D**). Furthermore, plants expressing the parasite target genes showed enhanced resistance to *P*. *aegyptiaca* as evidenced by abnormal parasite development and higher parasite mortality after attachment, as compared to non-transformed plants (**Figures 5A–C**). These results indicate that the resistance induced in lines 2, 17, and 59 through the use of hairpin silencing was considerable.

## Conclusion

In light of the importance of parasitic weeds to world agriculture and the difficulty of obtaining resistance by conventional methods, we assume that genetic resistance based on the silencing of key metabolic genes in the parasite is now feasible. We used different experimental systems and demonstrated that the TRV-VIGS system can provide a rapid screening process for the silencing of potential candidate parasite genes. In addition, the results of our work involving a hairpin-silencing strategy showed that short interfering RNA molecules expressed in host plants affect gene expression in parasitic plants attached to host roots. However, in this context, further research will be required to identify more gene sequences critical to the growth of the parasite and to optimize the system for siRNA signal transmission from host to parasite for use with other promoter sequences.

## Author Contributions

RA conceived, planned, and supervised the work. ND performed the molecular work and transgenic analysis. HE analyzed the data. DL helped in siRNA analysis. DW contributed in tissue culture and tomato transformation. JA-N, SM, and ME contributed in data production. AG-O contributed in gene constructs.

## Conflict of Interest Statement

The authors declare that the research was conducted in the absence of any commercial or financial relationships that could be construed as a potential conflict of interest.

## Footnotes

1 http://ppgp.huck.psu.edu

2 http://ppgp.huck.psu.edu/

3 http://ppgp.huck.psu.edu/
