# Supplementary material for: Enhanced Host-Parasite Resistance Based on Down-Regulation of Phelipanche aegyptiaca Target Genes Is Likely by Mobile Small RNA
Source: Front Plant Sci. 2017 Sep 12;8:1574. doi: 10.3389/fpls.2017.01574 (PMC5601039; doi:10.3389/fpls.2017.01574)
Supplement: Supplementary file 1 [file Data_Sheet_1.DOCX]

**Peroxidase (*PaPrx1*), 232bp**

CCAAGCAATTAAGTTTAGTGAAAAACAACAAATTTTCTGCTTTTTCATCAATATGGAGAACAAAAAATCCATCAACTTTGTTGCTGTGACGCTAGCAATATTATCACTCATACTCTTCCTGTCCAGCACACCAACTCAAGCACAACTATCTCCCACATTCTACTCTCGCACATGTCGTAATGCGCCAACTATAATTCGCAATTCCATCCGCAGAGCAATATCACGTGAGAGG

**Mannose 6-phosphate reductase (*PaM6PR*), 268bp**

TCCAATGAGGATATGGAACTGTTGAAGACTATGGAGCGGAAATACAGAACTAATCAACCTGCCAAGTTCTGGGGTATCGATCTTTTCGCATAAGTTTCTCTCCCATGTTAGGGATTTTCATTCATGTATGATGCAACACAAGGAGAGAGAGAGAGAGACGGAAAAGGAGTGTGCCATTTTATGGTAAAATAGTGTTTATTATTGCAGTGGGGCAATATTTCATCTACTTAAGAACTTAACTAAAACTGTATTGTTCTCTTCCAACCTC

***PaACS* synthase (*ACS*), 299bp**

TTGATGACGATCGAGTGGCGCATGGGTGGAACGTCAACCCATTCGCCGTCCTTGAGGAGCTGCAGGCCGCTGACCTTATCATCCTGGAAGAGAAGTATTATCCCGCCGGCATCGGTGTGGGCCCGAAGGCCCTTGATCAGATCGGGCTTGGGGCATGGTGGGTATTTGCTCACTTTGGTGCCAAAATTTGGGCCTGTGGTGCCATAGAATACCTTCTTCAGATAACCTTTCTCAAGCCCAAGATTCTCACATAGTAAATCCAGAAGCTGCTCCGCCAGTTTCTCCAGCTGGCCCGCAAA

**Supplementary Figure S1.** Sequences of the selected target-genes of interest. Three important genes of *P. aegyptiaca* (*PaPrx1*, *PaM6PR* and *PaACS*) were selected for knock-down. The sequences of *PaPrx1* (AY692263), *PaM6PR* (Aly *et al*., 2009) and *PaACS* (AB219097) of *P. aegyptiaca* were fished out from NCBI and confirmed with the PPGP website (<http://ppgp.huck.psu.edu>).


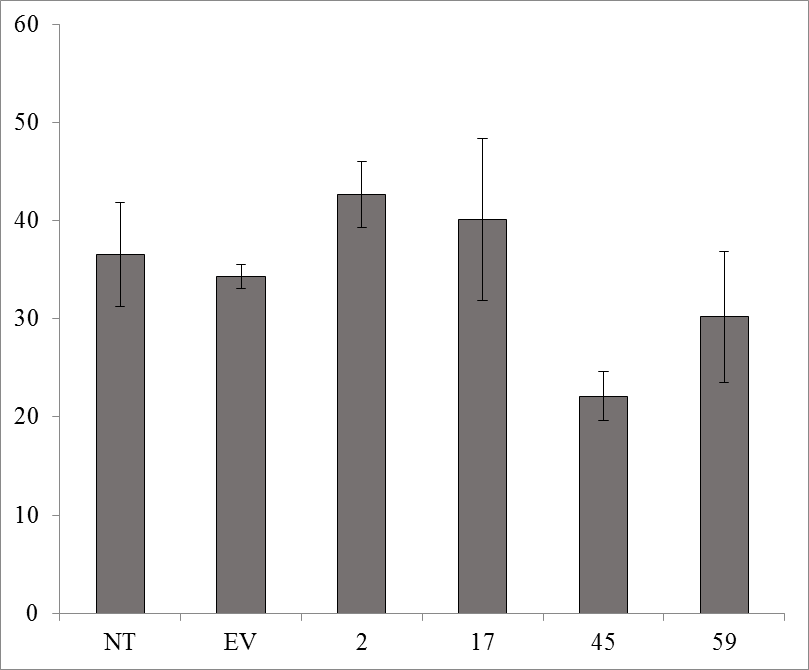


Tomato lines

**Supplementary Figure S2.** Measurement of peroxidase (*PaPrx1*) activity in the parasite tubercles.The peroxidase activity of 200 mg of the parasite tubercles attached to transgenic lines (2, 17, 45 and 59); empty victor (EV) and non-transgenic host roots (NT)wasassayed using theAmplex^®^ Red Hydrogen Peroxidase/Peroxidase Assay Kit (A22188, Molecular Probes).

| Similarity of sequences selected for peroxidase (*PaPrx1*) to sequencesfrom the http://ppgp.huck.psu.edu database | | | | |
| --- | --- | --- | --- | --- |
| **Stages of parasitic development of *Orobanche aegiptiaca*** | **Sequence ID** | **Score (bits)** | **E- Value** | **Identities** |
| Seed germination |  |  |  |  |
|  | OrAe0GB1_34611 | 321 | 9.00E-88 | 208/222 (93%) |
|  | OrAe0GB1_46289 | 182 | 5.00E-46 | 101/104 (97%) |
|  | OrAe0GB1_44959 | 182 | 5.00E-46 | 95/96 (98%) |
|  | OrAe0GB1_55785 | 172 | 5.00E-43 | 87/87 (100%) |
| Germinated seed, emerged radicle, pre-haustorial growth |  |  |  |  |
|  | OrAe1FB1_2210 | 460 | e-129 | 232/232 (100%) |
|  | OrAe1FB1_2542 | 420 | e-117 | 228/232 (98%) |
|  | OrAe1FB1_1415 | 412 | e-115 | 227/232 (97%) |
|  | OrAe1FB1_2227 | 410 | e-115 | 224/230 (97%) |
|  | OrAe1FB1_2777 | 406 | e-113 | 227/233 (97%) |
|  | OrAe1FB1_42817 | 404 | e-113 | 225/232 (96%) |
|  | OrAe1FB1_2395 | 383 | e-106 | 218/225 (96%) |
|  | OrAe1FB1_42543 | 373 | e-103 | 222/232 (95%) |
|  | OrAe1FB1_42482 | 373 | e-103 | 219/228 (96%) |
|  | OrAe1FB1_2583 | 365 | e-101 | 221/232 (95%) |
|  | OrAe1FB1_42622 | 311 | 6.00E-85 | 212/221 (95%) |
|  | OrAe1FB1_41276 | 297 | 1.00E-80 | 191/202 (94%) |
|  | OrAe1GB1_115999 | 178 | 1.00E-44 | 90/90 (100%) |
|  | OrAe1GB1_119348 | 139 | 1.00E-32 | 79/82 (96%) |
|  | OrAe1GB1_119421 | 135 | 2.00E-31 | 77/80 (96%) |
| Seedling after exposure to haustorial induction factors (HIFs) |  |  |  |  |
|  | OrAe2FB1_31889 | 452 | e-127 | 231/232 (99%) |
|  | OrAe2FB1_1312 | 420 | e-118 | 228/232 (98%) |
|  | OrAe2FB1_32324 | 412 | e-115 | 227/232 (97%) |
|  | OrAe2FB1_2384 | 392 | e-109 | 215/218 (98%) |
|  | OrAe2FB1_2252 | 369 | e-102 | 220/230 (95%) |
|  | OrAe2FB1_2389 | 361 | e-100 | 219/230 (95%) |
|  | OrAe2FB1_32178 | 351 | 7.00E-97 | 189/193 (97%) |
|  | OrAe2FB1_2689 | 325 | 4.00E-89 | 217/232 (93%) |
|  | OrAe2FB1_31810 | 299 | 2.00E-81 | 217/231 (93%) |
|  | OrAe2FB1_2537 | 299 | 2.00E-81 | 187/199 (93%) |
|  | OrAe2FB1_31106 | 202 | 4.00E-52 | 105/106 (99%) |
|  | OrAe2GB1_2511 | 100 | 3.00E-21 | 50/50 (100%) |
|  | OrAe2GB1_22339 | 84 | 2.00E-16 | 42/42 (100%) |
| Haustoria attached to host roots, penetration stages, pre-vascular connection (~48 h) |  |  |  |  |
|  | OrAe3GB1_86882 | 133 | 6.00E-31 | 70/71 (98%) |
|  | OrAe3GB1_48640 | 82 | 2.00E-15 | 44/45 (97%) |
| Early established parasite, parasite vegetative growth after vascular connection (~72 h) |  |  |  |  |
|  | OrAe41G2B1_76581 | 280 | 5.00E-75 | 175/185 (94%) |
|  | OrAe41G2B1_64562 | 172 | 8.00E-43 | 90/91 (98%) |
|  | OrAe41G2B1_56271 | 147 | 5.00E-35 | 77/78 (98%) |
| Pre-emergence from soil/roots |  |  |  |  |
|  | OrAe52FB1_854 | 444 | e-125 | 230/232 (99%) |
|  | OrAe52FB1_23394 | 400 | e-112 | 224/230 (97%) |
|  | OrAe52FB1_23443 | 371 | e-103 | 203/207 (98%) |
|  | OrAe52FB1_2618 | 226 | 1.00E-59 | 114/114(100%) |

**Supplementary Table S1.** Results of a nucleotide blast of the selected *PaPrx1* region with the transcriptomic data of *P. aegyptiaca* ESTs from the Parasitic Plant Genome Project (<http://ppgp.huck.psu.edu/>) at different developmental stages. In the sequence ID codes: **Or** indicates *Orobanche* plants; **Ae** indicates *aegyptiaca*; **0** indicates RNA from the stage of seed germination;**1**indicates RNA from germinated seed,the emerged radicle and pre-haustorial growth;**2**indicates RNA from seedlings after exposure to haustorial induction factors (HIFs); **3**indicates RNA from the stage of haustoria attaching to host roots, penetration stages and pre-vascular connection (~48 h);**41**indicatesRNA from the stage of the early established parasite and the parasite’s vegetative growth after the establishment of a vascular connection (~72 h), **52**indicates RNA from the stage of pre-emergence from soil/roots;**F**indicates Roche 454 FLX [average. 200–250 bp reads] -derived sequences;**G**indicatesIllumina GA2x, 81x81 bp paired-end sequences or sequences derived using similar methods;**B1**indicatesfirst-built data.
